# Is intravenously administered, subdissociative-dose KETAmine non-inferior to MORPHine for prehospital analgesia (the KETAMORPH study): study protocol for a randomized controlled trial

**DOI:** 10.1186/s13063-018-2634-3

**Published:** 2018-05-02

**Authors:** Clément Le Cornec, Said Lariby, Vivien Brenckmann, Jean Benoit Hardouin, Claude Ecoffey, Marion Le Pottier, Philippe Fradin, Hélène Broch, Amine Kabbaj, Yannick Auffret, Florence Deciron, Céline Longo, François Javaudin, Quentin Le Bastard, Joël Jenvrin, Emmanuel Montassier

**Affiliations:** 1 Emergency Department, Nantes University Hospital, 44000 Nantes, France; 2 Tours University Hospital, Emergency Medicine Department, Tours, France; 3 Emergency Department and Mobile Intensive Care Unit, CHU Grenoble Alpes, 38043 Grenoble Cedex 09, France; 4 SPHERE U1246, Inserm, université de Nantes-université de Tours, 44000 Nantes, France; 5 Department of Anaesthesia-Emergencies-Intensive Care and Internal Medicine and Geriatrics, Hôpital Pontchaillou, Université de Rennes 1, 2 Rue Henri Le Guilloux, 35033 Rennes Cedex, France; 6 Emergency Department, Centre Hospitalier Universitaire Angers, Angers, France; 7 Emergency Department, La Roche sur Yon Hospital, La Roche Sur Yon, France; 8 Emergency Department, Châteaubriant Hospital, Châteaubriant, France; 9 Emergency Department, Saint Nazaire Hospital, Saint Nazaire, France; 10 Quimper Hospital CHIC, Emergency Department SAMU, 29000 Quimper, France; 11 Emergency Department, Le Mans Hospital, Le Mans, France

**Keywords:** Ketamine, Morphine, Pain, Traumatic and non-traumatic

## Abstract

**Background:**

Acute pain is a common condition among prehospital patients and prompt management is pivotal. Opioids are the most frequently analgesics used in the prehospital setting. However, opioids are highly addictive, and some patients may develop opioid dependence, even when they are exposed to brief opioid treatments. Therefore, alternative non-opioid analgesia should be developed to manage pain in the prehospital setting. Used at subdissociative doses, ketamine, a noncompetitive *N-*methyl-D-aspartate and glutamate receptor antagonist, provides analgesic effects accompanied by preservation of protective airway reflexes. In this context, we will carry out a randomized controlled, open-label, multicenter trial to compare a subdissociative dose of ketamine to morphine to provide pain relief in the prehospital setting, in patients with traumatic and non-traumatic pain.

**Methods/design:**

This will be a multicenter, single-blind, randomized controlled trial. Consecutive adults will be enrolled in the prehospital setting if they experience moderate to severe, acute, non-traumatic and traumatic pain, defined as a numeric rating scale score greater or equal to 5. Patients will be randomized to receive ketamine or morphine by intravenous push. The primary outcome will be the between-group difference in mean change in numeric rating scale pain scores measured from the time before administration of the study medication to 30 min later.

**Discussion:**

This upcoming randomized clinical trial was design to assess the efficacy and safety of ketamine, an alternative non-opiate analgesia, to manage non-traumatic and traumatic pain in the prehospital setting. We aim to provide evidence to change prescribing practices to reduce exposition to opioids and the subsequent risk of addiction.

**Trial registration:**

ClinicalTrials.gov, ID: NCT03236805. Registered on 2 August 2017.

**Electronic supplementary material:**

The online version of this article (10.1186/s13063-018-2634-3) contains supplementary material, which is available to authorized users.

## Background

Pain is a common condition among prehospital patients [1]. In Australia, Jennings et al. reported that 34.5% of prehospital patients experienced pain, the majority presenting with traumatic or medical etiology (40.1% and 39.1%, respectively). Pain of a cardiac nature only accounted for 17.0% of presentations [2]. Rapid and efficient management of acute pain is pivotal in the prehospital setting. However, Jennings et al. found that a large percentage of patients arrived in the emergency department (ED) without significant pain reduction [2]. In France, Galinski et al. reported that, overall, 51% of the patients experienced pain relief during prehospital management, and that inadequate pain control is more frequent in patients with traumatic or gynecologic/obstetric pain [3].

Opioids are the most frequently prescribed analgesics in the prehospital setting [3, 4]. However, several issues should be highlighted. First, opioids are highly addictive, and some patients may develop opioid dependence, even if they are exposed to brief opioid treatments during in-hospital pain management [5–7]. Second, opioids prescription may be associated with severe adverse events, including oxygen desaturation and respiratory depression, hypotension, bradycardia, and oversedation, that may worse a patient’s condition [8, 9]. Other common acute side effects of opioids include dizziness, nausea, and vomiting [10].

Therefore, alternative non-opioid analgesia strategy, using agents at lower risk of dependence, should be proposed to manage pain in the prehospital setting [11]. Ketamine is a non-competitive *N-*methyl-D-aspartate and glutamate receptor antagonist that decreases central sensitization, “wind-up” phenomena, and pain memory [12–14]. Ketamine is commonly used at a dissociative dose for procedural sedation [15]. Used at a subdissociative dose (i.e., low-dose ketamine, 0.1 to 0.6 mg/kg and, most commonly, 0.3 mg/kg), ketamine provides analgesic effects, accompanied by preservation of protective airway reflexes, spontaneous respiration, and cardiopulmonary stability [14, 16, 17]. Relatively few studies have reported the use of low-dose ketamine alone for analgesia in the prehospital setting. Losvik et al. conducted a retrospective cohort study of trauma patients, in a low-cost rural trauma system in Iraq [18]. They reported that in patients with Injury Severity Score > 8, ketamine was associated with a significantly better effect on the systolic blood pressure compared to opioid analgesia (*p* = 0.03). Tran et al. performed a cluster randomized trial to compare the analgesic effects of ketamine and morphine in trauma patients, in a prehospital low-resource setting [19]. A total of 169 trauma patients were treated outside hospital settings with ketamine (administered as slow intermittent intravenous injections of doses of 0.2–0.3 mg/kg), while 139 patients were treated with morphine (administered in one single intramuscular dose of 10 mg for adult patients and 5 mg for child casualties). Visual Analogue Scale (VAS) ratings were measured by district physicians at the first in-field encounter before the administration of analgesic, and then by trained physicians and nurses at ED admission. The mean effect, as measured by VAS reduction, was 3.5 points for ketamine and 3.1 points for morphine (95% CI for a difference of − 0.8–0.09). The rate of vomiting was significantly lower in the ketamine group (5%) than in the morphine group (19%, 95% CI for difference 8–22%). The rate of hallucinations and agitation was higher in ketamine-treated patients (11%) than in the morphine-treated patients (1.5%, 95% CI for difference 4–16%).

To do methodological limitations of the previous studies, well-designed multicenter clinical studies to further examine the potential applicability and benefits of subdissociative-dose ketamine in the prehospital setting in trauma and non-trauma patients are needed. In this context, we will carry out a randomized, controlled, open-label multicenter trial to compare a subdissociative-dose ketamine alone to morphine alone to provide pain relief in the prehospital setting in patients with traumatic and non-traumatic pain. Here, we hypothesize that ketamine 20 mg, titrated during a 30-min period with an objective of verbal rating scale pain score of 3 or less, will provide non-inferior analgesia to morphine 3 mg, titrated during the same period, in a group of patients suffering moderate to severe pain in the prehospital setting.

## Methods/design

### Study design

**Fig. 1:**
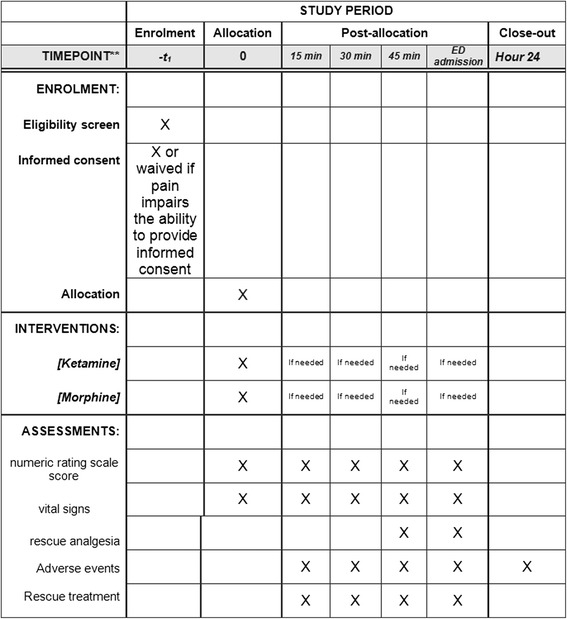
Standard Protocol Items: Recommendations for Interventional Trials (SPIRIT) Figure for the KETAMORPH trial. Schedule of enrollment, interventions, and assessments

This will be a multicenter, single-blind, randomized controlled trial to compare low-dose ketamine to morphine for analgesia in trauma and non-trauma patients in the prehospital setting. We perform a single-blind trial as side effects associated with ketamine can easily be observed (dizziness, mood change). Therefore, blinding may not be complete as it might be possible to determine arm during administration. Moreover, primary outcome will be assessed by the patient using the verbal rating scale, without any possible intervention of the physician in charge of the patient. The trial, named KETAMORPH, has been designed on the basis of the Consolidated Standards of Reporting Trials (CONSORT 2010) guidelines [20], and will be conducted in 10 hospitals in France, including five academic centers, as reported in Additional file 1. A SPIRIT Figure is provided (Fig. 1) and a SPIRIT Checklist is included as Additional file 2.

The study was supported by a grant from the French Ministry of Health (PHRC API16/N/059), sponsored by the Nantes University Hospital, and monitored by the Clinical Research Unit Grand Ouest. The study protocol and patient informed consent procedures were approved and received Sud-Méditerranée 2 Institutional Review Board approval (IRB sudmed 2, approval number 217 R26).

### Setting and study population

Consecutive adults (18 years or older) will be enrolled in the prehospital setting by emergency medicine services, if they experience moderate to severe, acute, non traumatic and traumatic pain, defined as a numeric rating scale score greater or equal to 5, on a standard 11-point (0: no pain, to 10: worst possible pain) numeric rating scale. The emergency medical services are ambulance base stations equipped with one or more mobile intensive care units, consisting of an ambulance driver, a nurse, and a senior emergency physician as the minimum team [21]. Exclusion criteria will be: unstable vital signs (systolic blood pressure < 90 or > 200 mmHg, pulse rate < 50 or > 150 beats/min, and respiration rate < 10 or > 30 breaths/min, Glasgow Coma Scale score < 15), pregnancy, breast-feeding, unable to give numeric rating scale scores, allergy to morphine or ketamine, acute pulmonary edema or acute heart failure, acute coronary syndrome or unstable ischemic heart disease, renal or hepatic insufficiency, patients who received morphine for the same acute pain or acute psychiatric illness, patients who require emergency fracture or joint reduction, head injury with acute intracranial hypertension, patient using buprenorphine, nalbuphine, pentazocine or naltrexone.

Informed consent may be waived at randomization, because patients will need urgent pain management and because acute pain impairs the ability to provide informed consent. Whenever a patient will be included without written informed consent, such consent will be promptly sought, according to the French Law of Ethics, subsequently from the patient when the pain has decreased [22]. Therefore, the senior emergency physician from the emergency medical service in charge of the patient will obtain informed consent once the patient has arrived in hospital. Then, a member of the research team of the prehospital and emergency department unit will follow the patient during the 24 h follow-up. A sample consent form is included as Additional file 3.

### Study protocol and intervention

Patients will be randomly assigned in a 1:1 ratio, using a computer-generated list to ketamine or morphine in the two groups of patients (i.e., traumatic and non-traumatic pain). Development of the randomization list, confirmation of written consent acquisition for all participants, and statistical analyses were conducted by the research manager and statistician, who were independent of any data collection. The randomization list was generated before commencement of the study. We used computer-generated random numbers to generate the allocation sequence, without blocking. The allocation sequence was then implemented in sealed envelopes, opened by the physician in charge of the patient.

Morphine will be administered by intravenous push, 2 mg (patient weight < 60 kg) or 3 mg (patient weight ≥ 60 kg) every 5 min, as recommended by the French guideline on acute pain management [23]. Ketamine will be administered by intravenous push of 20 mg followed by intravenous push of 10 mg every 5 min, as recommended by the French guideline on acute pain management [23]. Drugs will be administered until the patient has a pain with a rating scale score of less or equal to 3, or until the onset of a serious adverse event, or until ED admission. If a patient reports a pain numeric rating scale score of 5 or greater at 30 min, 45 min, 60 min or at ED admission, rescue analgesia will be administered to the patient for additional pain relief. The choice of drugs and dose will be left at the discretion of the emergency physician, as previously reported [24]. For patients with a blood oxygen saturation level (SpO_2_) below 94% during the procedure, oxygen will be administered with nasal cannulae-delivering flow rate of 2 L/min, and will be adapted based on SpO_2_ follow-up.

### Assessment of outcomes

The primary objective of the trial will to show that low-dose ketamine alone is not inferior to morphine alone at 30 min, in prehospital patients who experience moderate to severe, acute, traumatic or non-traumatic pain, defined as a numeric rating scale score greater or equal to 5. The primary outcome will be the between-group difference in mean change in verbal rating scale pain scores among patients receiving ketamine or morphine, measured from the time before administration of the study medication to 30 min later.

Secondary endpoints will be: (1) between-group difference in mean change in numeric rating scale pain scores among patients receiving ketamine or morphine, measured from the time before administration of the study medication to 15, 45, 60 min later, and at ED admission, (2) the incidence of rescue analgesia at 30, 45, and 60 min, and at ED admission, (3) the change in vital signs at 15, 45, 60 min and at ED admission, (4) the incidence of adverse events at 15, 45, 60 min and at ED admission, (5) the need to withdraw morphine or ketamine and the use of specific drugs to antagonize severe adverse events at 15, 45, 60 min and at ED admission, (6) weight based dose of study drug (mg/kg dosing) received during the 30-min period, and (7) number of doses of study drug received during the 30-min period. We will actively seek adverse events associated with morphine or ketamine use, including: oxygen desaturation and respiratory depression, hypotension, bradycardia, oversedation, dizziness, disorientation, mood change, nausea and vomiting, as previously reported [23]. Follow-up will end 24 h after the last administration of ketamine or morphine for each patient, based on the half-time elimination of the study drugs.

### Data collection

Prior to the trial initiation, study personnel will undergo training sessions on data collection and will be individually tested on data entry as well as outcome assessments. Study data will be collected and managed using Ennov clinical electronic data capture tools hosted at Nantes University Hospital. Ennov clinical is a secure, web-based application designed to support data capture for research studies, providing: (1) an intuitive interface for validated data entry; (2) audit trails for tracking data manipulation and export procedures; (3) automated export procedures for seamless data downloads to common statistical packages; and (4) procedures for importing data from external sources.

### Sample size under non-inferiority hypothesis

To assess non-inferiority in the two subgroups of patients (i.e., traumatic and non-traumatic pain), with a non-inferiority margin of 1.3, standard deviation (SD) of 3, α = 5%/2, β = 10%, 448 patients are needed (i.e., 112 in each group: morphine versus ketamine in traumatic patients, morphine versus ketamine in non-traumatic patients). These parameters are based on estimates of variability from previous works from Chang et al. [8, 24–26]. The most recent work used a between-group difference for change in mean pain score of 1.3 to define a statistically difference. Thus, we chose 1.3 to be the non-inferiority margin [24]. Considering 10% of non-evaluable subjects (refusal to participate in case of waived consent, death, ED admission before 30 min), 496 patients will be required.

### Statistical analysis

No interim analysis is planned.

Continuous variables will be summarized using descriptive statistics, i.e. number of subjects, mean, median, SD, interquartile range, and minimum and maximum. Qualitative variables will be summarized by frequency and percentage. Since this is a non-inferiority study, analysis of the primary outcome will be performed on a per-protocol population. Secondary analysis will be performed based on the intention-to-treat (ITT) principle. We will perform chi-square or Fisher’s exact tests as appropriate for qualitative variables, and the Mann-Whitney tests will be used for continuous variables. All statistical tests will be two-sided. The chosen type-I error rate will be α = 0.05. Analyses will be done using Stata software (Stata Corp, TX USA).

### Role of the funding source

The funding source will have no role in the study design, data collection, data analysis, data interpretation or writing of the report. All authors agreed to submit for publication.

## Discussion

This upcoming randomized clinical trial was design to assess the efficacy of ketamine, an alternative non-opioid analgesic to manage non-traumatic and traumatic pain in the prehospital setting. Pain is a common condition among prehospital patients [1]. Opioids are the drug of choice in the prehospital setting to manage moderate to severe, acute, non-traumatic and traumatic pain [3]. However, the choice of the analgesic to treat acute pain in prehospital patients lacks a clear evidence base, and changing prescribing practices is needed to reduce the number of patients exposed to morphine. This will limit adverse events associated with opioids use, especially the subsequent risk of addiction [24]. Indeed, risk of addiction was demonstrated in patients briefly exposed to opioids treatments in the ED [5, 11]. Moreover, Barnett et al. recently showed that long-term opioid use was significantly higher among patients treated by high-intensity ED opioid prescribers than among patients treated by low-intensity ED opioid prescribers [27].

Ketamine has the potential to decrease opioid use in the prehospital setting [11]. However, previous studies comparing low-dose ketamine to morphine for prehospital analgesia have methodological limitations, including retrospective design and evaluation of pain by the physician in charge of the patient [4, 19]. Indeed, Jennings et al. recommended that a well-designed randomized controlled trial with sufficient sample size and power should be developed to compare the analgesic efficacy of ketamine to opioids administered in the prehospital setting. They also recommend to compare the prevalence and magnitude of side effects, alterations in hemodynamic parameters, and variables reflecting oxygen balance (oxygen saturation or blood gas analysis when available) between ketamine and other analgesic agents [28].

The KETAMORPH trial should provide high-quality evidence to settle this issue in providing guidance on the use of ketamine in the prehospital setting to manage moderate to severe acute non-traumatic and traumatic pain. If it confirms the efficacy and the safety of the low-dose ketamine for analgesia in the prehospital setting, emergency physicians will have a lever to reduce opioid use and its addictive potential.

## Trial status

Recruiting. Recruitment began in November 2017 and is expected to conclude in May 2019. Target enrollment for the study is 498 participants. The trial is registered at ClinicalTrials.gov.

## Additional files

Additional file 1: Centers involved in the study. (DOCX 844 kb)

Additional file 2: SPIRIT Checklist. (DOC 121 kb)

Additional file 3: Consent form. (DOCX 127 kb)

## Abbreviations

CONSORT: Consolidated Standards of Reporting Trials
ED: Emergency department
ITT: Intention-to-treat

## References

[1] McLean SA, Maio RF, Domeier RM (2002). The epidemiology of pain in the prehospital setting. Prehospital Emerg. Care..

[2] Jennings PA, Cameron P, Bernard S (2011). Epidemiology of prehospital pain: an opportunity for improvement. Emerg Med J.

[3] Galinski M, Ruscev M, Gonzalez G, Kavas J, Ameur L, Biens D (2010). Prevalence and management of acute pain in prehospital emergency medicine. Prehospital Emerg. Care..

[4] Murphy AP, Hughes M, McCoy S, Crispino G, Wakai A, O’Sullivan R (2017). Intranasal fentanyl for the prehospital management of acute pain in children. Eur J Emerg Med.

[5] Bohnert ASB, Valenstein M, Bair MJ, Ganoczy D, McCarthy JF, Ilgen MA (2011). Association between opioid prescribing patterns and opioid overdose-related deaths. JAMA.

[6] Herzig SJ, Rothberg MB, Cheung M, Ngo LH, Marcantonio ER (2014). Opioid utilization and opioid-related adverse events in nonsurgical patients in US hospitals. J Hosp Med.

[7] Brennan MJ, Stanos S (2010). Strategies to optimize pain management with opioids while minimizing risk of abuse. PM R.

[8] Chang AK, Bijur PE, Napolitano A, Lupow J, Gallagher EJ (2009). Two milligrams i.v. hydromorphone is efficacious for treating pain but is associated with oxygen desaturation. J Opioid Manag.

[9] Motov S, Rosenbaum S, Vilke GM, Nakajima Y (2016). Is there a role for intravenous subdissociative-dose ketamine administered as an adjunct to opioids or as a single agent for acute pain management in the emergency department?. J Emerg Med.

[10] Benyamin R, Trescot AM, Datta S, Buenaventura R, Adlaka R, Sehgal N (2008). Opioid complications and side effects. Pain Physician.

[11] Butler MM, Ancona RM, Beauchamp GA, Yamin CK, Winstanley EL, Hart KW (2016). Emergency department prescription opioids as an initial exposure preceding addiction. Ann Emerg Med.

[12] Guirimand F, Dupont X, Brasseur L, Chauvin M, Bouhassira D (2000). The effects of ketamine on the temporal summation (wind-up) of the R(III) nociceptive flexion reflex and pain in humans. Anesth Analg.

[13] Schmid J, Tortorano AM, Jones G, Lazzarini C, Zhang N, Bendall MJ (2011). Increased mortality in young candidemia patients associated with presence of a *Candida albicans* general-purpose genotype. J Clin Microbiol.

[14] Motov S, Rockoff B, Cohen V, Pushkar I, Likourezos A, McKay C (2015). Intravenous subdissociative-dose ketamine versus morphine for analgesia in the emergency department: a randomized controlled trial. Ann Emerg Med.

[15] Svenson JE, Abernathy MK (2007). Ketamine for prehospital use: new look at an old drug. Am J Emerg Med.

[16] Galinski M, Dolveck F, Combes X, Limoges V, Smaïl N, Pommier V (2007). Management of severe acute pain in emergency settings: ketamine reduces morphine consumption. Am J Emerg Med.

[17] Smith DC, Mader TJ, Smithline HA (2001). Low dose intravenous ketamine as an analgesic: a pilot study using an experimental model of acute pain. Am J Emerg Med.

[18] Losvik OK, Murad MK, Skjerve E, Husum H (2015). Ketamine for prehospital trauma analgesia in a low-resource rural trauma system: a retrospective comparative study of ketamine and opioid analgesia in a ten-year cohort in Iraq. Scand J Trauma Resusc Emerg Med.

[19] Tran KP, Nguyen Q, Truong XN, Le V, Le VP, Mai N (2014). A comparison of ketamine and morphine analgesia in prehospital trauma care: a cluster randomized clinical trial in rural Quang Tri province, Vietnam. Prehospital Emerg Care.

[20] Schulz KF, Altman DG, Moher D, CONSORT Group (2010). CONSORT 2010 Statement: updated guidelines for reporting parallel group randomised trials. Trials.

[21] Adnet F, Lapostolle F (2004). International EMS systems: France. Resuscitation.

[22] Jabre P, Combes X, Lapostolle F, Dhaouadi M, Ricard-Hibon A, Vivien B (2009). Etomidate versus ketamine for rapid sequence intubation in acutely ill patients: a multicentre randomised controlled trial. Lancet.

[23] Vivien B, Adnet F, Bounes V, Chéron G, Société française d’anesthésie et de réanimation (SFAR), Société française de médecine d’urgence (2010). Sedation and analgesia in the emergency context. Ann Fr Anesth Reanim.

[24] Chang AK, Bijur PE, Esses D, Barnaby DP, Baer J (2017). Effect of a single dose of oral opioid and nonopioid analgesics on acute extremity pain in the emergency department: a randomized clinical trial. JAMA.

[25] Chang AK, Bijur PE, Holden L, Gallagher EJ (2015). Comparative analgesic efficacy of oxycodone/acetaminophen versus hydrocodone/acetaminophen for short-term pain management in adults following ED discharge. Acad Emerg Med.

[26] Chang AK, Bijur PE, Munjal KG, John GE (2014). Randomized clinical trial of hydrocodone/acetaminophen versus codeine/acetaminophen in the treatment of acute extremity pain after emergency department discharge. Acad Emerg Med.

[27] Barnett ML, Olenski AR, Jena AB (2017). Opioid-prescribing patterns of emergency physicians and risk of long-term use. N Engl J Med.

[28] Jennings PA, Cameron P, Bernard S (2011). Ketamine as an analgesic in the pre-hospital setting: a systematic review. Acta Anaesthesiol Scand.

