# Supplementary material for: Is intravenously administered, subdissociative-dose KETAmine non-inferior to MORPHine for prehospital analgesia (the KETAMORPH study): study protocol for a randomized controlled trial
Source: Trials. 2018 May 2;19:260. doi: 10.1186/s13063-018-2634-3 (PMC5930801; doi:10.1186/s13063-018-2634-3)
Supplement: Supplementary file 1 — Centers involved in the study. (DOCX 844 kb) [file 13063_2018_2634_MOESM1_ESM.docx]

| **Hospital and Town** | **Principal investigator** |
| --- | --- |
| Nantes University Hospital, Nantes | Clément Le Cornec |
| Tours University Hospital, Tours | Said Lariby |
| Grenoble University Hospital, Grenoble | Vivien Brenckmann |
| Rennes University Hospital, Rennes | Claude Ecoffey |
| Angers University Hospital, Angers | Marion Le Pottier |
| La Roche sur Yon Hospital, La Roche sur Yon | Philippe Fradin |
| Châteaubriant Hospital, Châteaubriant | Hélène Broch |
| Saint Nazaire Hospital, Saint Nazaire | Amine Kabbaj |
| Quimper Hospital, Quimper | Yannick Auffret |
| Le Mans University Hospital, Le Mans | Florence Deciron |
