# Supplementary material for: Is intravenously administered, subdissociative-dose KETAmine non-inferior to MORPHine for prehospital analgesia (the KETAMORPH study): study protocol for a randomized controlled trial
Source: Trials. 2018 May 2;19:260. doi: 10.1186/s13063-018-2634-3 (PMC5930801; doi:10.1186/s13063-018-2634-3)
Supplement: Supplementary file 3 — Consent form. (DOCX 127 kb) [file 13063_2018_2634_MOESM3_ESM.docx]

| 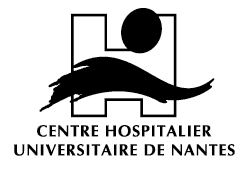 | **Formulaire de consentement pour la poursuite de l’étude**  **« Kétamine versus Morphine pour la prise en charge de la douleur aiguë en pré hospitalier : essai randomisé contrôlé »**  **Promoteur*: CHU de Nantes***  **Réf : RC17_0082 (Ketamorph) *N° EudracT / n° d’enregistrement : 2017-000930-69*** |
| --- | --- |
| **Version n° …….**  **Date : ……………** |  |

Je soussigné(e)

M^e^, M^lle^, M. *(rayer les mentions inutiles*) (*prénom*, NOM) *………….….….…………*………………………………………… …………….…………………………………………………………………………………………………………………………,

Date de naissance : …../……/……..

**accepte librement et volontairement de poursuivre ma participation à la recherche** **référencée ci-dessus,** coordonnée par le Docteur Joel Jenvrin et organisée par le CHU de Nantes, promoteur de la recherche.

**Etant entendu que :**

- Cette recherche m’a été clairement expliquée par oral et par écrit. Il m'a été remis une note d'information sur cette recherche précisant son but, sa méthodologie, les informations sur les traitements utilisés dont l’un m’a été administré en urgence pour soulager ma douleur et ce qu’il m’est actuellement demandé.
- Le médecin qui m’a informé(e) et a répondu clairement à toutes mes questions, m’a précisé que ma participation est libre et que je peux refuser d’y participer, cela quelles que soient mes raisons. Le fait de ne pas participer à cette recherche ne portera pas atteinte à mes relations avec ce médecin, ni à la qualité des soins qui me seront donnés.
- J’accepte que les personnes en charge du suivi de la recherche et astreintes au secret professionnel, aient accès aux données de mon dossier médical.
- J’accepte que les données enregistrées à l’occasion de cette recherche puissent faire l’objet d’un traitement informatisé par le promoteur ou pour son compte. Ces données seront identifiées par un numéro de code et mes initiales pour préserver mon anonymat. J’ai bien noté que le droit d’accès prévu par la CNIL (loi du 6 janvier 1978 modifiée relative à l’informatique, aux fichiers et aux libertés (art.39)) s’exerce à tout moment auprès du médecin qui me suit dans le cadre de la recherche et qui connaît mon identité. Je pourrai exercer mon droit de rectification et d’opposition auprès de ce même médecin, qui en informera le promoteur de la recherche.
- J’accepte l’utilisation de mes données à des fins de communications / publications sous format anonyme.
- Si, je le souhaite, à son terme, je serai informé(e) par le médecin des résultats globaux de cette recherche.
- J’atteste ne pas faire l'objet de mesure de protection (tutelle, curatelle, sauvegarde de justice), en outre je confirme être affilié(e) ou bénéficier d'un régime de sécurité sociale.
- Je ne pourrai pas participer à une autre recherche biomédicale pendant les 24 heures suivant ma prise en charge par le médecin urgentiste de l’équipe mobile de la Smur.
- Je suis parfaitement conscient(e) que je peux retirer à tout moment mon consentement à ma participation à cette recherche et cela quelles que soient mes raisons et sans supporter aucune responsabilité, mais je m’engage dans ce cas à en informer le médecin. Le fait de ne plus participer à cette recherche ne portera pas atteinte à ma prise en charge médicale.
- Le présent consentement ne dégage pas le promoteur et les investigateurs de leurs responsabilités.
- J’accepte que mon médecin traitant puisse être informé de ma participation à la recherche :

Oui, j’accepte Non, je refuse

| Date : | *Le cas échéant* : Attestation du consentement en cas d’impossibilité d’expression écrite de la personne qui se prête à la recherche biomédicale (précision du titre de la personne) | |
| --- | --- | --- |
| Signature du patient : | Date :  Prénom NOM : | Signature : |

| Signature du médecin qui atteste avoir pleinement expliqué à la personne signataire le but, les modalités ainsi que les risques potentiels de la recherche. | |
| --- | --- |
| Date : | NOM et Signature : |

Ce document est à réaliser en 2 exemplaires originaux : le premier doit être conservé par l’investigateur et le deuxième est remis à la personne donnant son consentement. En cas de duplicata, l’original est conservé par l’investigateur et une copie est remise à la personne ayant donné son consentement. En cas de triplicata, le promoteur récupèrera les consentements dans des enveloppes scellées tout au long de l’étude.
